# Effect of *Curcuma zedoaria* hydro-alcoholic extract on learning, memory deficits and oxidative damage of brain tissue following seizures induced by pentylenetetrazole in rat

**DOI:** 10.1186/s12993-020-00169-3

**Published:** 2020-10-06

**Authors:** Touran Mahmoudi, Zahra Lorigooini, Mahmoud Rafieian-kopaei, Mehran Arabi, Zahra Rabiei, Elham Bijad, Sedigheh Kazemi

**Affiliations:** 1 Medical Plants Research Center, Basic Health Sciences Institute, Shahrekord University of Medical Sciences, Shahrekord, Iran; 2 Department of Biology, Shahrekord University, Shahrekord, Iran; 3 Cellular and Molecular Research Center, Basic Health Sciences Institute, Shahrekord University of Medical Sciences, Shahrekord, Iran

**Keywords:** *Curcuma zedoaria*, Pentylenetetrazole, Repeated seizures, Oxidative stress, Memory

## Abstract

**Background:**

Previous studies have shown that seizures can cause cognitive disorders. On the other hand, the *Curcuma zedoaria* (CZ) has beneficial effects on the nervous system. However, there is little information on the possible effects of the CZ extract on seizures. The aim of this study was to investigate the possible effects of CZ extract on cognitive impairment and oxidative stress induced by epilepsy in rats.

**Methods:**

Rats were randomly divided into different groups. In all rats (except the sham group), kindling was performed by intraperitoneal injection of pentylenetetrazol (PTZ) at a dose of 35 mg/kg every 48 h for 14 days. Positive group received 2 mg/kg diazepam + PTZ; treatment groups received 100, 200 or 400 mg/kg CZ extract + PTZ; and one group received 0.5 mg/kg flumazenil and CZ extract + PTZ. Shuttle box and Morris Water Maze tests were used to measure memory and learning. On the last day of treatments PTZ injection was at dose of 60 mg/kg, tonic seizure threshold and mortality rate were recorded in each group. After deep anesthesia, blood was drawn from the rats’ hearts and the hippocampus of all rats was removed.

**Results:**

Statistical analysis of the data showed that the CZ extract significantly increased the tonic seizure threshold and reduced the pentylenetetrazol-induced mortality and the extract dose of 400 mg/kg was selected as the most effective dose compared to the other doses. It was also found that flumazenil (a GABA_A_ receptor antagonist) reduced the tonic seizure threshold compared to the effective dose of the extract. The results of shuttle box and Morris water maze behavioral tests showed that memory and learning decreased in the negative control group and the CZ extract treatment improved memory and learning in rats. The CZ extract also increased antioxidant capacity, decreased MDA and NO in the brain and serum of pre-treated groups in compared to the negative control group. Conclusion: It is concluded that the CZ extract has beneficial effects on learning and memory impairment in PTZ-induced epilepsy model, which has been associated with antioxidant effects in the brain or possibly exerts its effects through the GABAergic system.

## Background

Excessive neuronal excitability and excessive production of free radicals play an important role in the pathogenesis of a significant range of neurological disorders, including epilepsy. Increased susceptibility of the brain to oxidative damage highlights the importance of understanding the role of oxidative stress in the pathophysiology of seizures and epilepsy [1]. In addition, the evidence gathered demonstrates the importance of oxidative stress as a result of epileptic seizures. Long-term seizures have been found to be associated with oxidative damage to lipids, DNA, and proteins. It has also been well established that oxidative damage plays an important role in the pathogenesis and complications of other CNS disorders such as learning and memory disorders [2].

Defects in GABAergic inhibitory transmission are a prominent feature of temporal lobe epilepsy and have been demonstrated in animal models of epilepsy. GABAergic inhibition involves phasic and tonic inhibitions that are mediated by synaptic and outside synaptic GABA_A_ receptors, respectively. Rodent models of temporal lobe epilepsy (TLE) are associated with changes in expression and function of the GABA receptor in the hippocampus [3].

GABA receptors are target sites for drugs such as benzodiazepines. Classical benzodiazepines exhibit their therapeutic effects by bonding to the benzodiazepine site of the GABA receptor, which causes chloride flow through the ion channel complex, which has a wide range of side effects [4]. Since antiepileptic drugs have to be used for a long time, sometimes for a lifetime, and on the other hand, the use of chemical drugs has side effects and sometimes drug poisoning and these side effects limit their use. As a result, it does not achieve the desired therapeutic effect.

*Curcuma zedoaria Rosc* is also known as white turmeric, a perennial rhizomatous plant belonging to the family Zingiberaceae. It has been traditionally used to treat menstrual disorders, indigestion, vomiting and cancer [5]. Phytochemical studies show that all extracts of this plant have a percentage of terpenoids, alkaloids, saponins, flavonoids, glycosides and carbohydrates, phenols, tannins and phytosterols. It is also a rich source of essential oils, starches, curcumin and gums. Compounds such as curcumin, tetrahydro-methoxy curcumin, tetrahydro-bismethoxy curcumin were separated from two acetic-aqueous root extracts with two sesquiterpene [5].

The kindling model has become a widely employed technique for studying seizure mechanisms and considered to be a useful experimental model for human epilepsy. Pentylenetetrazole (PTZ) kindling is an acknowledged model for epilepsy and refers to a phenomenon in which repeated injection of a convulsant causes gradual seizure development culminating in generalized tonic–clonic seizures associated with a cognitive deficit. Examination of PTZ-kindled rat brains also revealed a significant neuronal cell loss in hippocampal CA1 and CA3 structures and the hilus, possibly the cause of observed cognitive deficits [6].

In the present study, the protective effects of CZ extract on the severity of epileptic seizures and memory disorders in rats kindled with pentylenetetrazole were investigated. It is hoped that the herb can be used along with other available medicines to treat epilepsy as a natural or low-risk medication if it is acceptable.

## Material and methods

### Plant collection

The rhizome of the CZ was purchased from a reputable local grocery and was approved by the botanist and registered with Herbarium No. 178 at the Medicinal Plants Research Center of Shahrekord University of Medical Sciences.

### Extraction

After purchasing the CZ rhizome, it was powdered with mill and extracted by a 70% ethanol or maceration method with a 5: 1 ratio, respectively. Erlenmeyer content of the plant was placed on a magnetic stirrer and its contents were smoothed after 72 h. Rotary apparatus was used for evaporation of water and ethanol of clarified solution and the incubator at 37 °C was used for final drying of concentrated extract [7].

### Determination of antioxidant potential of CZ extract by DPPH method

The extract was first prepared at 1 mg/ml concentration and DPPH at 0.1 mmol (dissolved in ethanol). Using the obtained stocks, six concentrations of 5 to 100 Mg including 10, 20, 40, 60, 80, and 100 are prepared for extract in a volume of 2 ml. 2 ml of DPPH was added to each of the six concentrations of the extract and placed in the dark for 15 min. A control tube containing 2 ml of ethanol and 2 ml of DPPH is prepared next to the samples. After 15 min, the absorbance of blank and the samples were read with spectrophotometer. Using the formula below, the IC50 is calculated [6].

$$ I(\% ) = 100 \times \frac{{A_{control} - A_{sample} }}{{A_{control} }} $$

### Determination of total phenolic content of the extract

Total phenolic compounds were measured according to Folin-ciocaltue colorimetric method and gallic acid. Briefly, 0.5 ml of the extract solution (0.01 g in 10 ml of 60% methanol) and 0.5 ml of Folin Ciocalteu solution were added and after 3–5 min, 0.4 ml of 7.5% sodium carbonate was added. After 30 min incubation at room temperature, the absorbance of the samples was read against distilled water blanks. Simultaneously with the experiment, different dilutions of gallic acid were prepared and as described above, standard curve was prepared; absorbance of samples was compared with standard curve and total phenol content of extract was calculated in mg of gallic acid per gram of dry extract [8].

### Determination of total flavonoid content of the extract

Briefly, 0.5 ml of the extract solution (0.01 g in 10 ml of 60% methanol) was mixed with 0.5 ml of 2% aluminum chloride and 3 ml of 5% potassium. After 40 min, the absorbance of the samples was read against distilled water at 415 nm. At the same time, different dilutions of rutin were prepared and tested as described above and standard curve was prepared. Absorbance of the samples was compared with the standard curve and the flavonoid content of each extract was calculated in milligrams of rutin per gram of dried extract [9].

### Laboratory animals

PTZ (Sigma Aldrich) was freshly prepared daily, and the study groups were injected every 48 h for 14 days (first at 35 mg/kg and last day at 60 mg/kg). Last day in all groups after injection of 60 mg/kg PTZ, tonic seizure threshold and mortality rate were recorded and compared. PTZ can be used to develop both acute and chronic animal models of epilepsy. For example, an acute injection of PTZ in rodents at a threshold dose (60–100 mg/kg, i.p. or s.c.) leads to myoclonic jerks, clonus, and tonic extension. However, repeated administration of PTZ at lower sub-threshold doses (20 to 40 mg/kg, i.p.) may induce kindling phenomena [10].

Treatment with CZ extract was performed daily. At the end of the 14th day after the seizure results were recorded, the animals were subjected to deep anesthesia and blood was collected for biochemical studies. Hippocampal tissue was also removed and transferred to the freezer at −70 °C until the day of the experiment. Also for histological examination, hippocampal tissue was fixed in 10% formaldehyde.

### Animal groups

The number of rats used in this study was 70. Adult male Wistar rats were maintained at 200–250 g under appropriate temperature (21 ± 2) and 12 h light and 12 h dark with free access to equal water and food. The animals were divided into the following groups for testing:

Negative control group: Animals (n = 10) received distilled water daily and PTZ at a dose of 35 mg/kg intraperitoneally every 48 h.

Sham group: Animals (n = 10) received normal saline intraperitoneally daily.

Diazepam group: Animals (n = 10) received diazepam at 2 mg/kg intraperitoneally 30 min before PTZ injection at 35 mg/kg.

CZ extract group: Animals (n = 10) received the CZ extract at 100, 200, 400 mg/kg intraperitoneally 30 min before PTZ injection at 35 mg/kg.

Effective dose of CZ with Flumazenil group: Animals (n = 10) received Flumazenil at the dose of 0.5 mg/kg after 30 min of injection of effective dose of the extract and then received PTZ at dose of 60 mg/kg intraperitoneally.

### Behavioral tests

#### Spatial memory test

For this test, rats were given 60 s to locate the platform in each attempt, whereas the rats were directed to the platform if they did not find the platform. The rats were given 30 s rest between the two trials to examine the surroundings. Rats were removed from the water for approximately 10 min between the blocks and rested in cages. Each rat was trained four days, four times daily, and the fifth day was the probe day. The experiment was performed once without the platform [11]. This test was performed 30 min after receiving the extract.

#### Passive avoidance test

The test was performed for each rat for four days. On the first and second test days, each rat was put into the apparatus for five minutes. On the third day an acquisition test was performed. Rats were individually housed in the bright room. After a matching period (2 min), the guillotine lid was opened, and after the rat entered the dark chamber the lid was closed and an electric shock was provided to the animal (1 mA, 1 s, and 1 time). In this test, the initial delay in entering the dark room was recorded on the second day, and the delay time while passing was recorded on the third day [12]. The animals received the extract 30 min before the start of the experiment.
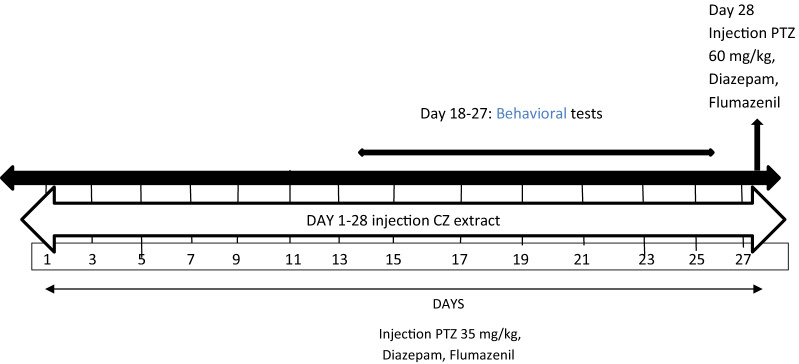


### Biochemical analysis

#### Measurement of antioxidant capacity of serum and brain tissue

Measurement of antioxidant capacity of serum and brain tissue was evaluated by FRAP method. This method was based on the ability of serum and brain homogenate tissue to recover Fe^3+^ (ferric) to Fe^2+^ (ferrous) ions in the presence of TPTZ2 reagent, resulting in the blue complex of TPTZ-Fe^2+^ with a maximum absorbance of 593 nm [13].

#### Measurement of serum and brain tissue malondialdehyde (MDA)

200 µl of serum/homogenate brain tissue was mixed with 1.5 ml of 20% acetic acid, 1.5 ml of TBA (0.8%) and 200 µl of 8.1% SDS solution. The samples were then boiled in Bain Marie for 60 min. The samples were then cooled and 1 ml of distilled water and 5 ml of n-butanol-pyridine mixture were added and shaker. The mixture was then centrifuged at 4000 rpm for 10 min and the optical absorbance of the supernatant was recorded at 523 nm [13].

#### Measurement of nitric oxide (NO) content in serum and brain tissue

The procedure was performed by pouring 100 µl of each serum / tissue homogenate sample (in triplicate) into a 96-well plate. 100 µl of sulfanilamide solution (one gram of sulfanilamide in 100 cc phosphoric acid 5%) was added. The plate was incubated in the dark at room temperature for 5–10 min. 50 µl of NEDD * solution was added to wells and incubated at room temperature for half an hour, then light absorbance at 540 nm was read by ELISA reader and determined by standard nitrite curve in samples [13].

### Histopathological examination

The animal's brain was removed from the skull and placed in a 10% fixative solution. After a few days, samples were subjected to dewatering, clarification, paraffin immersion and finally dominance. After dominance, 5 μm thick sections were prepared by microtome and stained with hematoxylin eosin (H&E). These slices were then examined and photographed using light microscopy.

### Measurement of gamma-aminobutyric acid type A receptor alpha4 subunit gene expression

Samples of rat brain were frozen in an adequate volume of acid guanidium thyocianate solution and kept at −80 °C until RNA extraction. Total cellular RNA was extracted by the method of acid guanidium thyocianate phenol/chloroform extraction. Total tissue RNA concentration was measured by spectrophotometric s the quality of isolated RNA was verified by agarose gel electrophoresis with ethidium bromide staining. One microgram of purified total RNA was used as substrate for reverse transcription. The reaction was performed by incubation of RNA with 1 µM oligo(dT) and 200 units of MMLV reverse transcriptase from a Clontech first strand cDNA synthesis kit. An aliquot (5 µl of a 1/10 dilution) of the cDNA of each sample was used for RT-PCR. The PCR primers used was shown in Table 1. DNA amplification was carried out in 1 × Taq polymerase buffer, 1.5 mM MgCI2 supplemented with 50 µM dNTP, 0.25 µM of 5′ and 3′-specific primers, 1 µCi of [α-32p] and 2 units of Taq polymerase (Promega C) in a final volume of 50 µl. The mixture was overlaid with mineral oil and amplified for 30 cycles (each consisting of denaturation for 1 min at 94 °C, annealing for 1 min at 60 °C, extension for 1 rain at 72 °C) then extension for 7 min at 72 °C and storage at 4 °C in a Triothermoblock. Ten µl of cDNA products were size-separated by electrophoresis on a 10% acryl/bisacrylamide gel and stained with ethidium bromide (15 µg/ml). Each band was excised from the gel and the quantity of 32p incorporated was measured in a scintillation counter.

**Table 1:** The PCR primer's sequences

Gens	*Forward*	*Reverse*
Β actin	GCTCTCTTCCAGCCTTCCTTCCT	CATCCTGTCAGCAATGCCTGGGT
GABA_A_ R4α	CAGACATATATCCCGTGCATCA	CAGACAGCTATGAACCAATCCA

### Data analysis

Data were analyzed using Prism6 and Excel software using one way ANOVA, Tukey posttest and chi-square test. The difference between the groups was considered significant with at least p < 0.05. Results were reported as Mean + SEM.

## Results

### Antioxidant potency by DPPH method

CZ hydroalcoholic extract showed antioxidant activity in inhibiting DPPH radicals. The concentration of the extract that inhibited 50% of DPPH radicals (IC50) was calculated 279.78 μg/ml.

### Phenolic and flavonoid content

The content of total phenolic compounds of the hydroalcoholic extract of the CZ was 12.07 mg gallic acid per gram of extract. The content of total flavonoid compounds of hydroalcoholic extract was 19.43 mg rutin per gram of extract.

### Tonic seizure threshold (Phase IV)

**Fig. 1:**
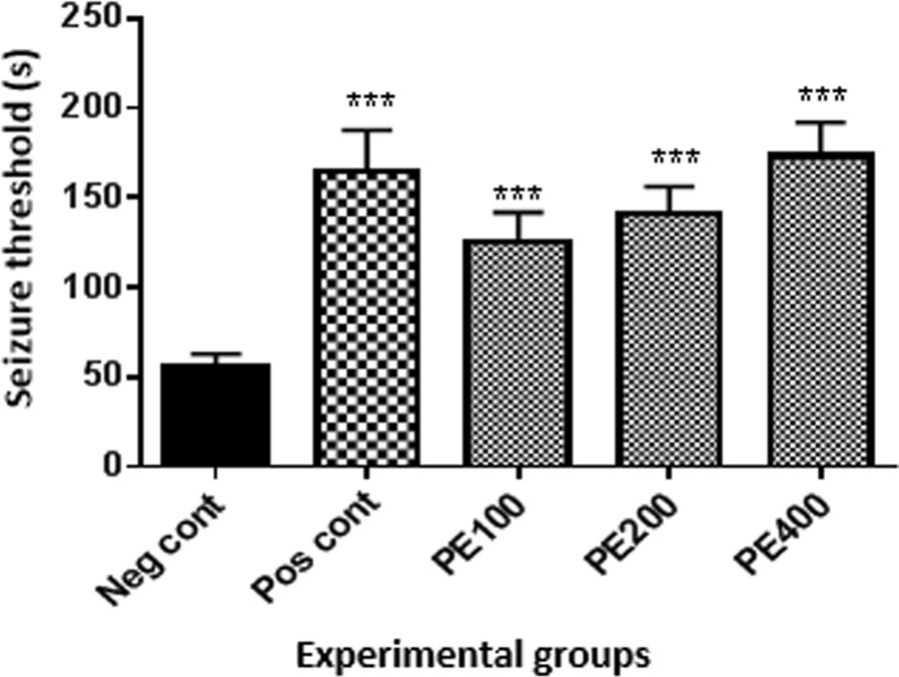
Effect of hydroalcoholic extract of CZ rhizome on tonic seizure threshold (Phase IV): Neg cont: vehicle (distilled water) + PTZ recipient group; Pos cont: diazepam + PTZ recipient group; PE 100, 200 or 400 CZ extract at 100, 200 or 400 mg/kg + PTZ recipient groups. *Comparison of groups with negative control (***at p < 0.001)

According to Fig. 1, the threshold of tonic seizure in the positive control group was significantly higher than the negative control group (p < 0.001). Tonic seizure threshold was significantly higher in the groups received the extract at concentrations of 100, 200 and 400 mg/kg plus PTZ than the negative control group (p < 0.001).

### The effect of flumazenil on tonic seizure threshold

**Fig. 2:**
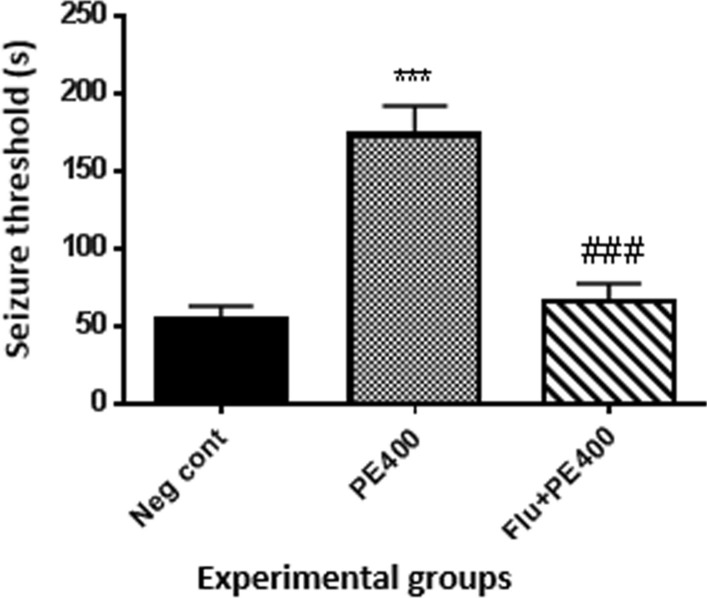
Effect of flumazenil on tonic seizure threshold (phase IV) in the group received the effective concentration of the CZ extract: Flu + PE400: the group received flumazenil plus CZ extract at 400 mg/kg + PTZ. *In compared to the negative control group (***at p < 0.001). ^#^ In compared to the PE400 group (^###^at p < 0.001)

According to Fig. 2, the tonic seizure threshold in the group received flumazenil with 400 mg/kg CZ extract and PTZ was lower than the group received 400 mg/kg of CZ extract with PTZ (p < 0.001).

### Mortality rate

**Fig. 3:**
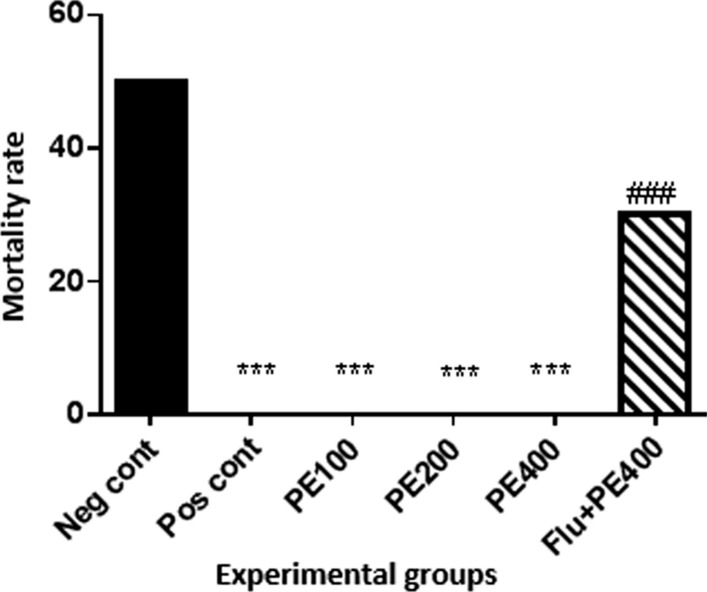
Effect of hydroalcoholic extract of CZ on mortality rate *Comparison of groups with negative control group (***at p < 0/001). ^#^In compared to the group received the CZ extract at 400 mg/kg with PTZ (^###^p < 0.001)

Chi-square analysis showed that there was a significant difference between different groups (p < 0.05). According to Fig. 3, data analysis showed that the mortality rate in the positive control group was zero and there was a significant difference with the negative control group (p < 0.001). The mortality rate in the groups received the extract at 100, 200 and 400 mg/kg plus PTZ was zero and there was a significant difference with the negative control group (p < 0.001). There was a significant difference in mortality rate between the group received flumazenil with the extract at 400 mg/kg and PTZ and the group received the extract at 400 mg/kg alone (p < 0.001).

### Spatial learning

**Fig. 4:**
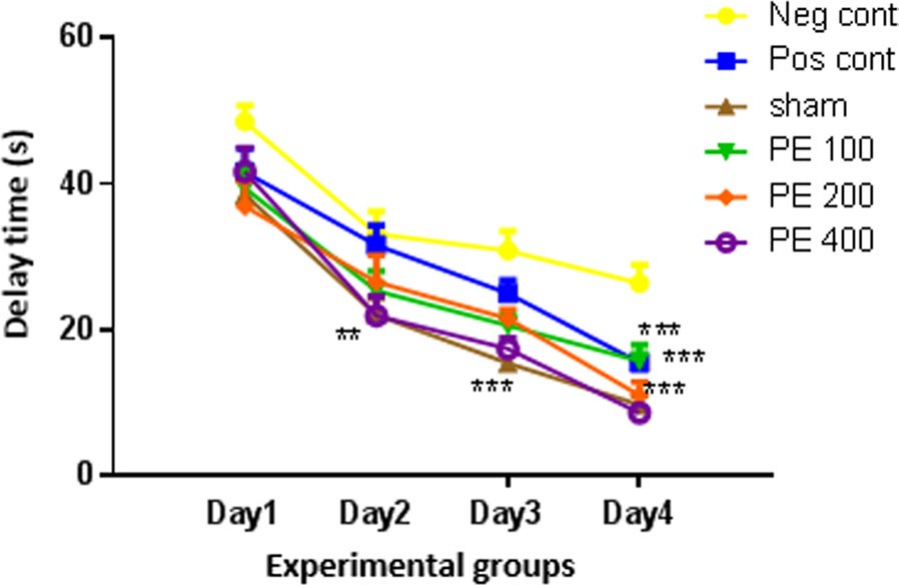
The effect of CZ hydroalcoholic extract on spatial learning in the first to fourth days *In compared to the negative control group (*at p < 0.05, **at p < 0.01, ***at p < 0.001)

According to Fig. 4, the latency of finding the platform in sham group was significantly less than that of negative control group (p < 0.001). Delay time of finding the platform on day 2 was significantly lower in the group received the extract at 400 mg/kg plus PTZ than the negative control group (p < 0.01). On the fourth day, the delay in finding the platform in the positive control group and the groups received the CZ extract at concentrations of 100, 200 and 400 mg/kg was significantly lower than the negative control group (p < 0.01).

### Spatial memory on probe day

**Fig. 5:**
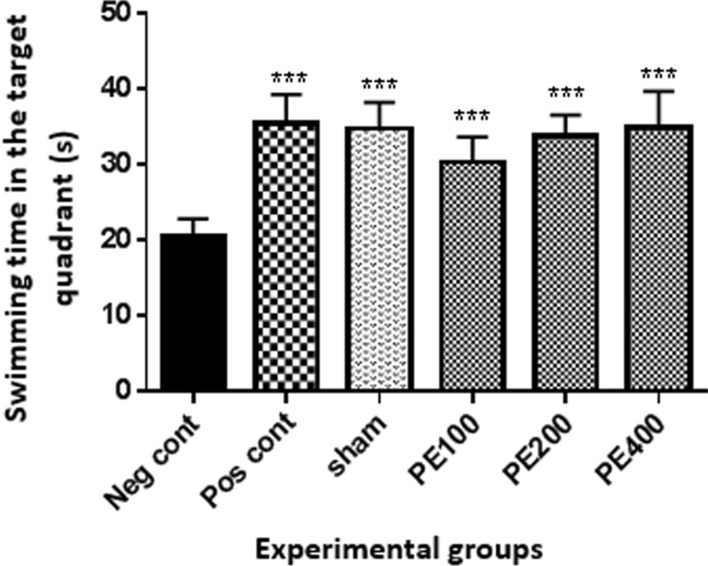
The effect of CZ hydroalcoholic extract on spatial learning on probe day. *In compared to the negative control group (***at p < 0/001)

The effect of intraperitoneal injection of different doses of the CZ extract on swimming time in the target quadrant is shown in Fig. 5. Swimming time in the target quadrant in all groups was significantly longer than the negative control group (p < 0.001).

### Passive avoidance memory

**Fig. 6:**
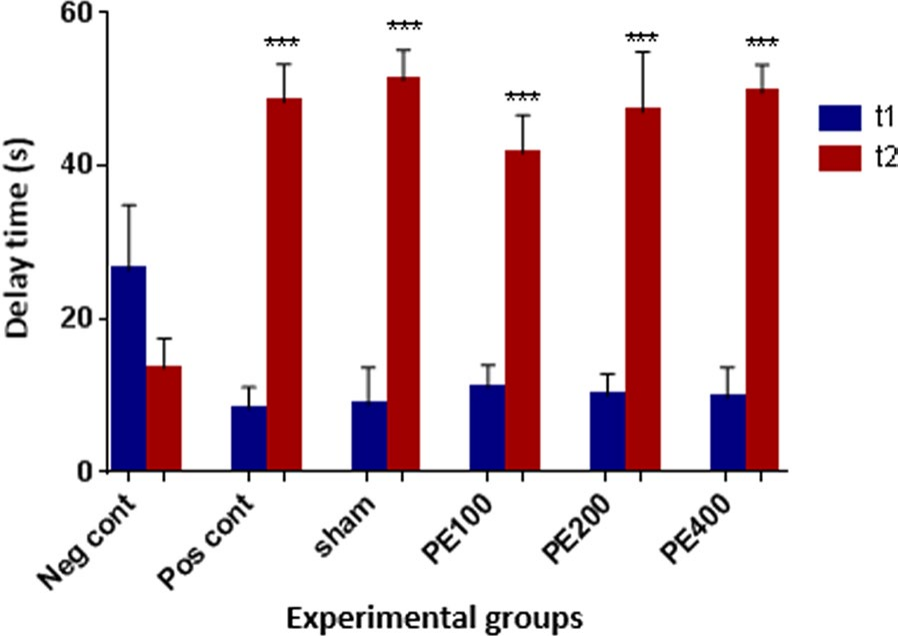
The effect of the CZ extract on the passive avoidance memory. *In compared to the negative control group (***at p < 0/001)

The effect of intraperitoneal injections of different concentrations of the CZ extract on the comparison of primary and secondary latencies related to avoidance memory is shown in Fig. 6. The results showed that the secondary delay time in all groups was significantly higher than the negative control group (p < 0.001).

### Antioxidant capacity of serum

**Fig. 7:**
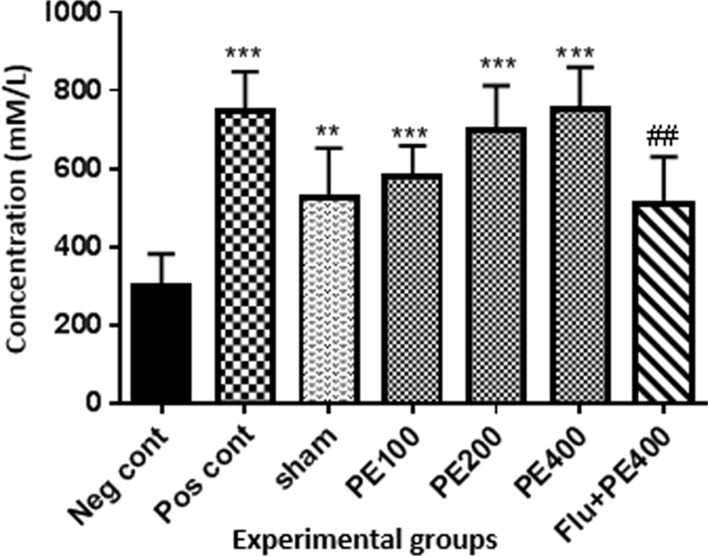
The effect of CZ extract on serum antioxidant capacity. *In compared to the negative control group (**at p < 0.01 and ***at p < 0.001). #: in compared to the group received the effective concentration of extract plus PTZ (^##^at p < 0.05)

According to Fig. 7, the antioxidant capacity of serum in the positive control group and the groups received extract at 100, 200 and 400 mg/kg plus PTZ was higher than the negative control group (p < 0.001).

### Antioxidant capacity of brain tissue

**Fig. 8:**
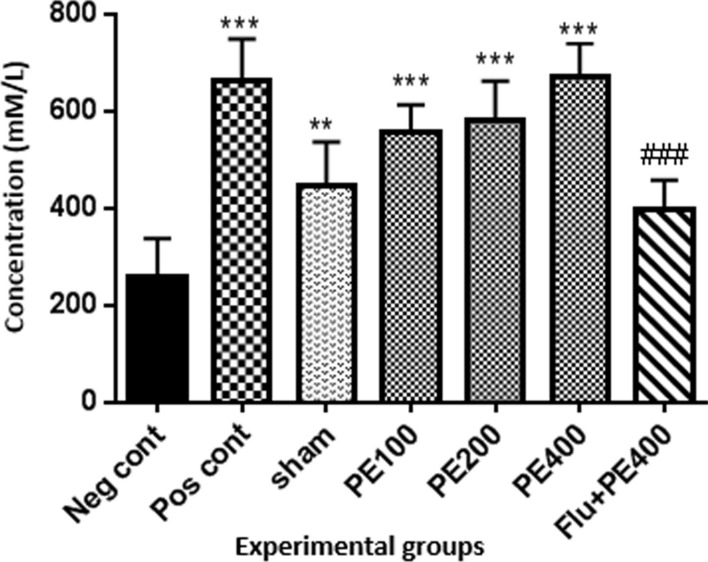
Effect of CZ extract on antioxidant capacity of the brain. *In compared to the negative control group (***at p < 0.001, **at p < 0.05). ^#^In compared to the group received the effective concentration of extract plus PTZ (^###^at p < 0.001)

According to Fig. 8, the antioxidant capacity of the brain in the positive and control group and the groups received CZ extract (at 100, 200 and 400 mg/kg) plus PTZ was higher than the negative control group (p < 0.001). Also in the sham group, in compared with the negative control group, there was a significant increase in TAC level of brain tissue (p < 0.01). The antioxidant capacity of the group received flumazenil with extract (at 400 mg/kg) and PTZ was significantly lower than the group received effective dose of extract (400 mg/kg) with PTZ (p < 0.001).

### Serum MDA levels

**Fig. 9:**
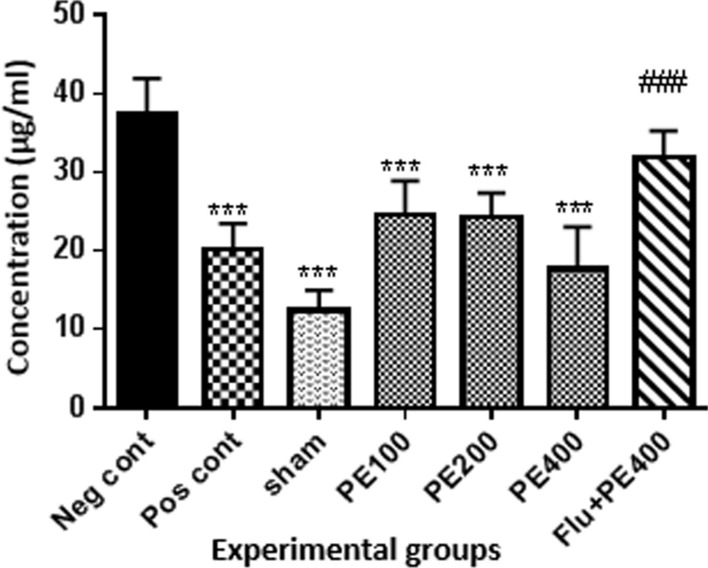
The effect of CZ extract on serum MDA. *In compared to the negative control group (***at p < 0.001). ^#^In compared to the group received the effective concentration of extract plus PTZ (^###^at p < 0.001)

According to Fig. 9, serum MDA levels in the positive control, sham and extract (100, 200 and 400 mg/kg plus PTZ) groups were significantly lower than in the negative control group (p < 0.001). MDA levels in the group received flumazenil with the extract (at 400 mg/kg) and PTZ were significantly higher than the group received the extract (at 400 mg/kg) with PTZ (p < 0.001).

### Brain tissue MDA levels

**Fig. 10:**
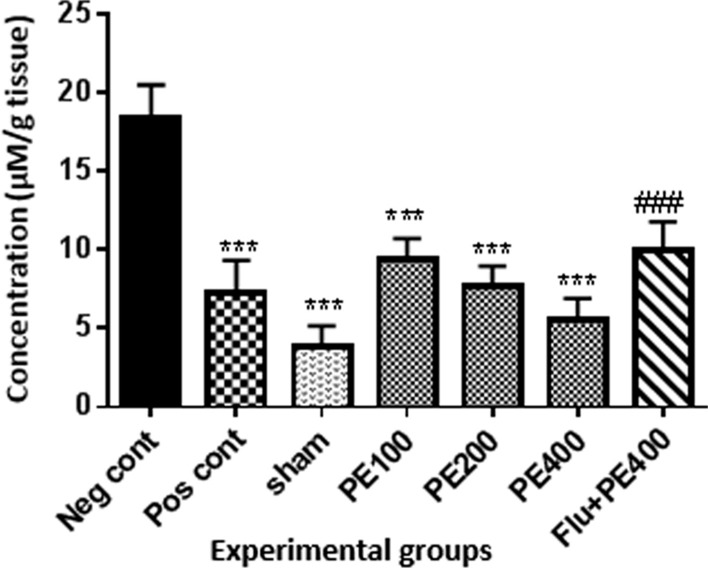
The effect of CZ extract on brain MDA. *In compared to the negative control group (***at p < 0.001). ^#^In compared to the group received the effective concentration of extract plus PTZ (^###^at p < 0.001)

According to Fig. 10, brain MDA content in the positive control, sham and extract (at 100, 200 and 400 mg/kg plus PTZ) groups was lower than the negative control group (p < 0.001). MDA levels in the group received flumazenil with the extract (400 mg/kg) and PTZ were significantly higher than the group received the extract (400 mg/kg) with PTZ (p < 0.001).

### Serum NO levels

**Fig. 11:**
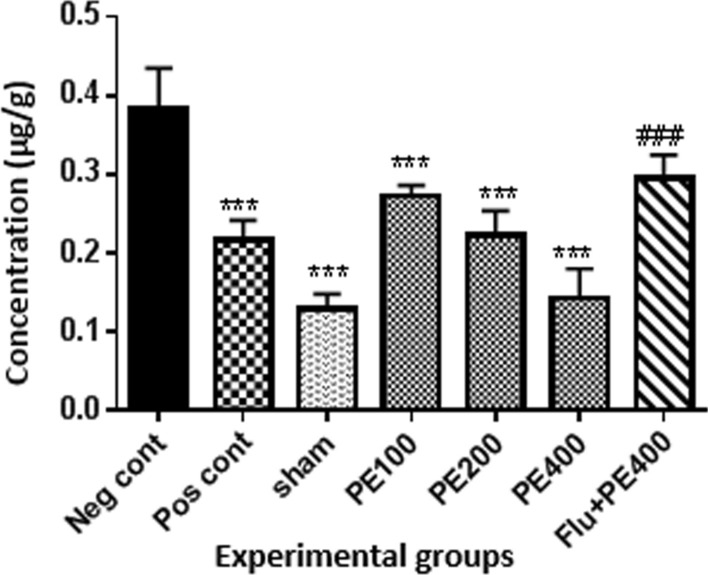
Effect of CZ extract on serum nitric oxide. *In compared to the negative control group (***at p < 0.001). ^#^In compared to the group received the effective concentration of extract plus PTZ (^###^at p < 0.001)

The results (Fig. 11) showed that serum nitric oxide levels were lower in the positive control, sham and extract (100, 200 and 400 mg/kg plus PTZ) groups than in the negative control group (p < 0.001). The nitric oxide level in the group received flumazenil with the extract (400 mg/kg) and PTZ was significantly higher than the group received the extract (400 mg/kg) with PTZ (p < 0.001).

### Brain tissue NO levels

**Fig. 12:**
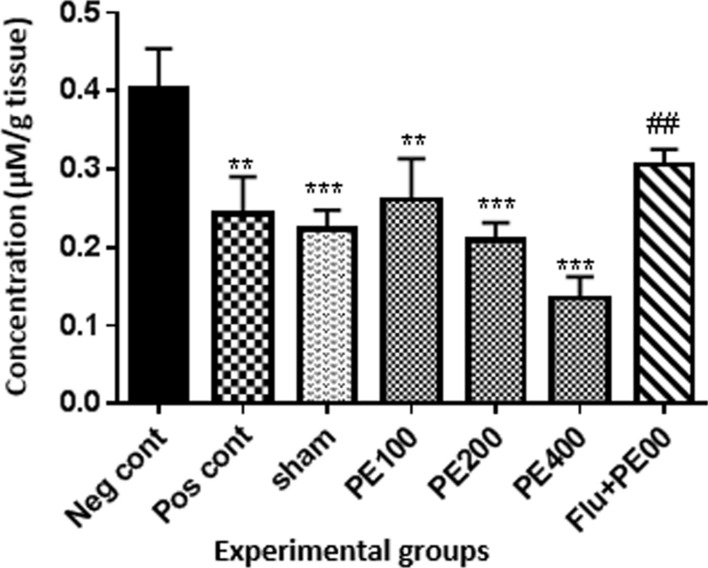
Effect of CZ extract on nitric oxide levels in brain tissue. *In compared to the negative control group (**at p < 0.01 and ***at p < 0.001). ^#^In compared to the group received the effective concentration of extract plus PTZ (^##^at p < 0.05)

Results (Fig. 12) showed that brain tissue nitric oxide levels were lower in the positive control, sham and extract (200 and 400 mg/kg plus PTZ) groups than in the negative control group (p < 0.001). The nitric oxide level in the group received flumazenil with the extract (400 mg/kg) and PTZ was significantly higher than the group received the extract (400 mg/kg) with PTZ (p < 0.05).

### Histological changes in the hippocampus

The following figures show the tissue pathology of all groups. In microscopic observations, hippocampal tissue is intact in the sham group. In hippocampal tissue samples, the number of pyramidal cells in the negative control group decreased and was absent in some areas. Hippocampal tissue samples in the positive control group lost a number of granular cells in the dentate gyrus. Molecular area cells, especially small cells in the CA1 and CA2 areas, decreased and the gliosis increased.

In the extract recipient group at 100 mg/kg plus PTZ, a number of small pyramidal cells in the CA1 and 2 regions were removed. No significant decrease in cell density in CA3 and 4 was observed in the group received the extract at a concentration of 200 mg/kg plus PTZ. In the group received the extract at 400 mg/kg with PTZ, the molecular layer of nerve cells decreased as well as granular cells in the dentate area. In the group received the extract at concentration of 400 mg/kg with PTZ and flumazenil, cellular density disappeared in the granular area.


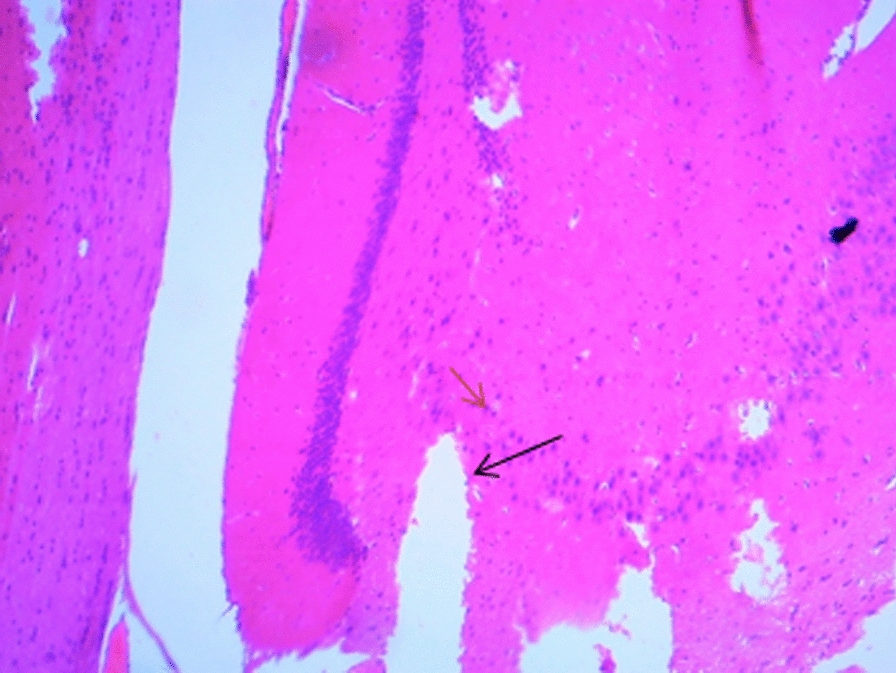
Figure A-1. Hippocampal structure in the PTZ group. a: Decrease in the number of pyramidal cells, b: No pyramidal cells.


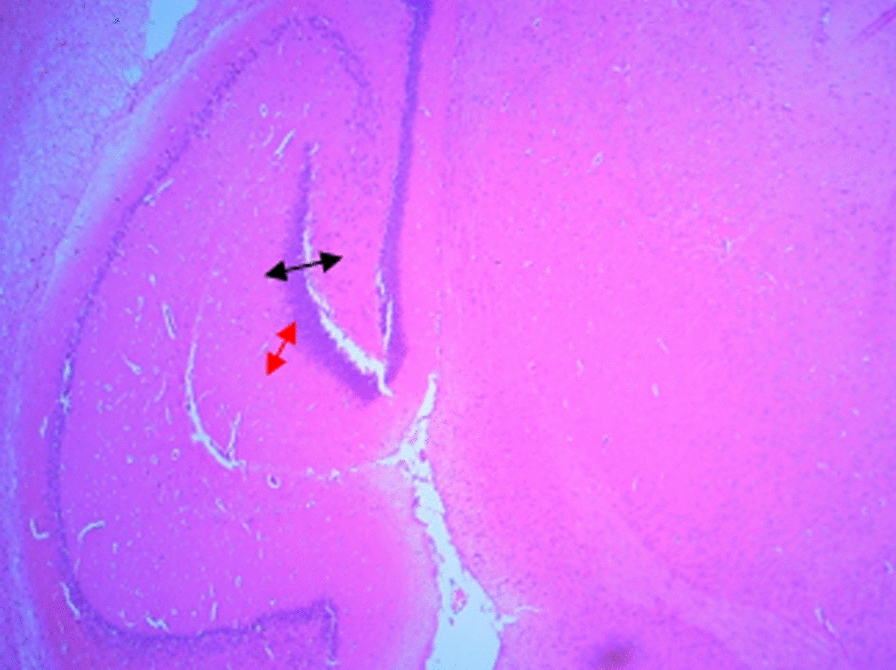
Figure 1-B. Hippocampal structure in sham group: Layer and cells are normal. a: granular layer, b: molecular layer.


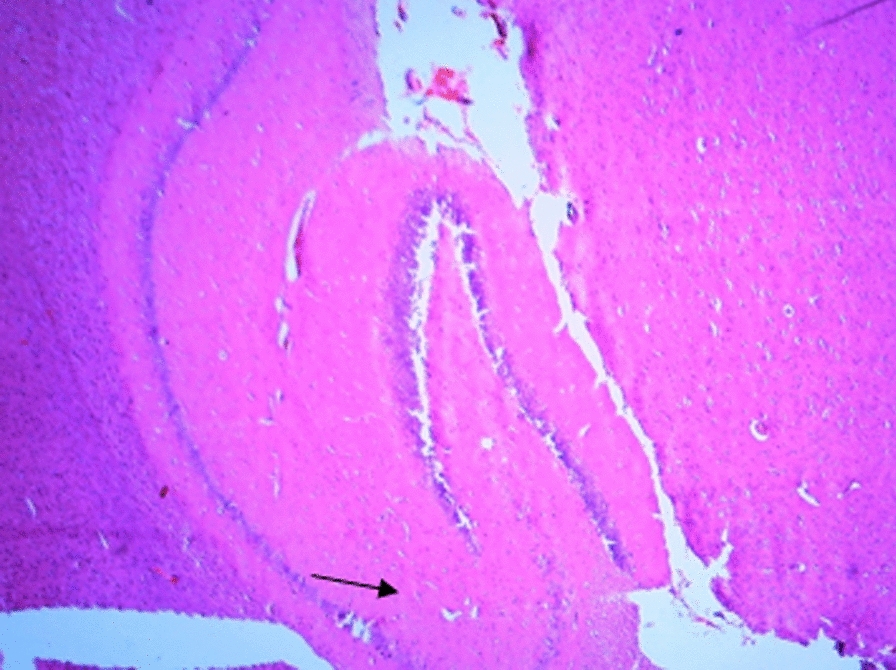
Figure 1-C. Hippocampal structure in the extract group (100 mg/kg). a: Destruction of small pyramidal cells in CA1 and 2 regions.


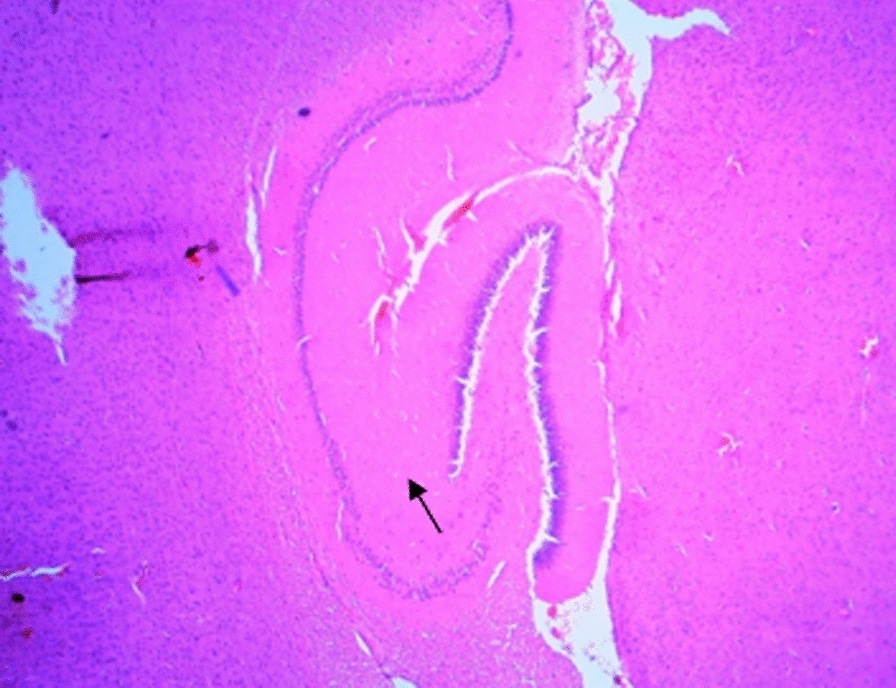
Figure 1-D. Hippocampal structure in the extract group (200 mg/kg). a: Cellular density decrease in CA3 and 4 regions.


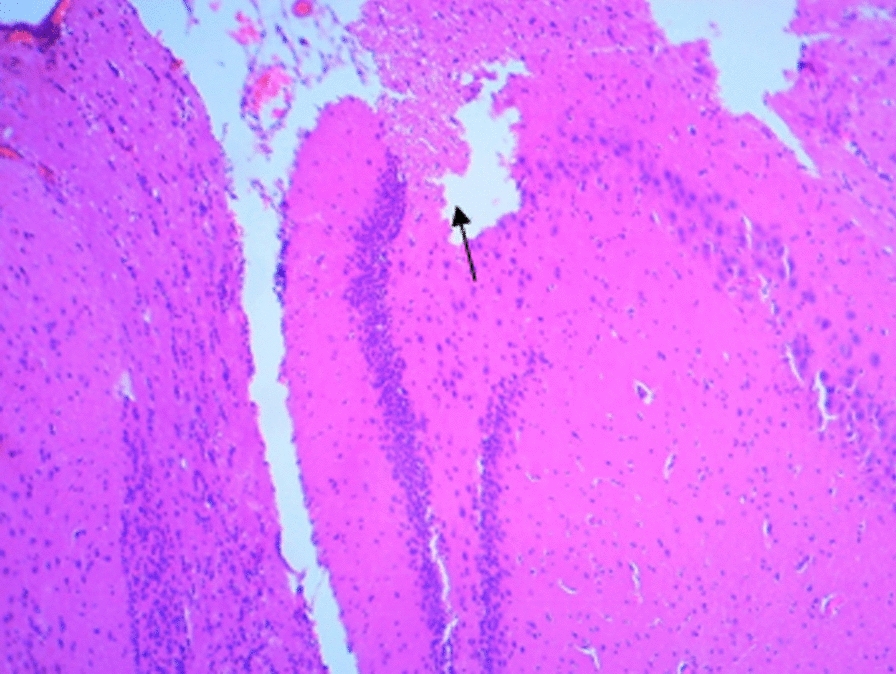
Figure 1-E. Hippocampal structure in the extract group (400 mg/kg). a: Decrease of granular cells in the dentate area.


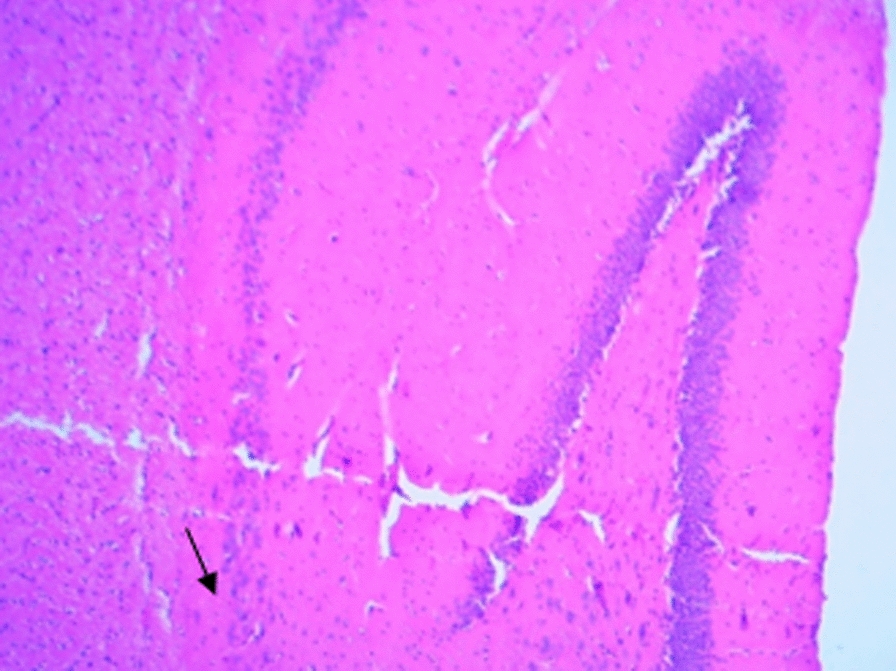
Figure 1-F. Hippocampal structure in the extract + Flumazenil group: loss of cellular density in the granular area.

### Expression of GABA_A_ receptor subunit 4α gene

**Fig. 13:**
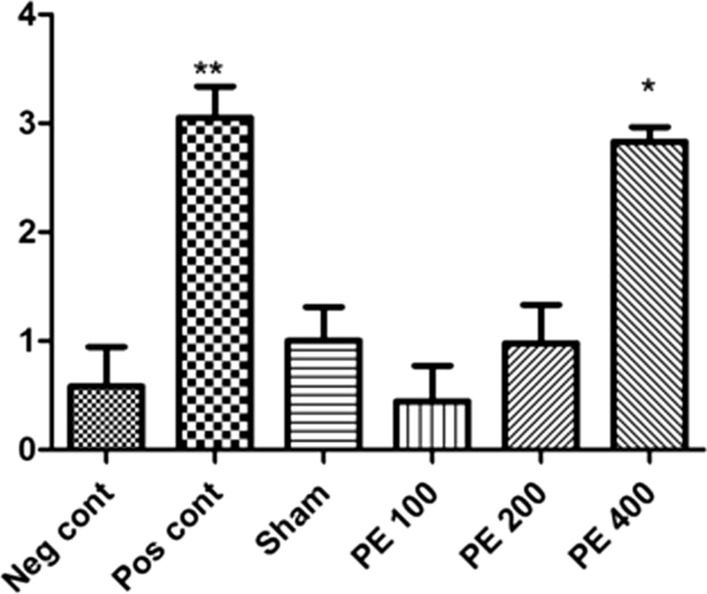
Effect of different doses of CZ extract and diazepam on GABA_A_ receptor 4α subunit mRNA expression. *In compared to the negative control group (*at p < 0.05 and **at p < 0.01)

The results show that the expression of GABA_A_ receptor subunit 4α gene was significantly increased in the diazepam group in compared to the negative control group. The 400 mg/kg CZ extract significantly increased GABA_A_ receptor subunit 4α gene expression (Fig. 13).

## Discussion

In the present study, the protective effects of CZ extract on the severity of epileptic seizures and memory disorders in PTZ-induced rats were investigated. Due to the fact that PTZ at a dose of 60 mg/kg leads to death with tonic contractions and generalized tonic–clonic attacks, this dose was used to induce seizures. Studies have shown that PTZ can act by blocking GABA_A_ receptors in the central nervous system neurons [14]. A research has shown that PTZ increases intracellular calcium ion concentrations and NMDA receptors are involved in this increase. On the other hand, it has been shown that an increase in calcium in the cell prevents the inhibitory effects of GABA [15]. In the present study, hydroalcoholic extract of CZ was able to increase tonic seizure threshold and decrease mortality rate in PTZ induced kindling model. Doses of 100, 200 and 400 mg/kg of extract completely inhibited PTZ induced death. Diazepam was used as a positive control to compare the anticonvulsant potency of the plant extract. It has been found that diazepam is an antagonist of PTZ induced seizures by increasing GABA neurotransmitters [16]. CZ extract at 400 mg/kg had a stronger effect on seizure control than diazepam, probably due to the presence of flavonoid compounds such as curcumin. Flumazenil, a specific GABA_A_ receptor antagonist, was also used to investigate the mechanism of the effect of the extract on PTZ-induced kindling. The results showed that it reduced the anticonvulsant effect of the effective dose of the plant extract in rats. Therefore, one of the main mechanisms involved in its anticonvulsant effect may be stimulation of the GABAergic system and benzodiazepine receptor. One of the basic mechanisms of anticonvulsant activity is activation of the GABAergic inhibitory system and benzodiazepines play an essential role in the stabilization of the nervous system [17]. Given this important feature of benzodiazepines, it can significantly inhibit seizures if components of the CZ have benzodiazepine receptor-stimulating properties.

The results of this study showed that the CZ extract at dose of 400 mg/kg significantly increased GABA_A_ receptor subunit 4α gene expression. In a 1998 study by Syu et al., Compounds such as curcumin, tetrahydrodymethoxy curcumin and tetra-tetrahydro bis-dimethoxy curcumin were extracted as the most important flavonoids found in the extract of the CZ [18]. Anovadiya et al. (2013) showed that curcumin can inhibit PTZ-induced seizures by activating the brain's benzodiazepine-GABAergic system and antioxidant activity [19].

Convulsive seizures lead to impaired memory and learning in the individual. The incidence of learning, memory and cognitive impairment in experimental animal models of epilepsy has been previously confirmed [20]. It has been found that in epilepsy induced by intraperitoneal injection of PTZ (Kindling method) and administration at increasing doses, neurons involved in learning and memory in the hippocampus (CA1, CA2 and dentate) are damaged and the same is true for some amygdala neurons [20]. This may justify some of the learning and memory disorders in epilepsy. In addition, epileptic seizures and seizure attacks exacerbate oxidative stress in the brain, including in the hippocampus, which is one of the major causes of neuronal degeneration and learning and memory deficits in epileptic mice [21]. The direct role of flavonoids in the acquisition, memory, recording, and storage of memory has previously been described in a study involving neuronal signaling activation and gene expression in the brain [22]. The results of the behavioral tests showed that seizures in male Wistar rats decreased spatial memory and passive avoidance memory, whereas pretreatment with CZ extract improved spatial and passive avoidance memories following seizures in rats. Also, the time elapsed and the distance traveled in the target quadrant were higher in the Morris water maze test in all extract-treated groups than the PTZ (negative control) group. On the beneficial effects of curcumin on learning and memory in passive avoidance testing and the Y-maze test in the study by Scaini et al. 2012, it has been shown that antioxidants due to oxygen free radical scavenging ability improve learning and memory [23]. And curcumin probably exerted some of its beneficial effects on learning and memory in this way. In addition, another part of the beneficial effect of curcumin in the 2010 study by Yadav et al., may be attributed to the strengthening of the cholinergic system in the presence of this antioxidant [24].

Reactive oxygen species are produced during seizures and are involved in seizure-induced neural cell death. Recent studies show that oxidative stress and mitochondrial dysfunction can make the brain prone to epileptic seizures. On the other hand, researches have shown that seizures lead to the production of free radicals and oxidative damage to proteins, fatty acids, and nucleic acids in cells, therefore, oxidative stress and the production of free radicals are now recognized as both the cause and the product of seizure attacks [25]. In this regard, antioxidants decrease the severity and likelihood of seizures by reducing the oxidative stress and lipid peroxidation caused by increased oxygen free radical formation in areas involved in the pathogenesis of epilepsy including the hippocampus [26]. Antioxidants are able to increase neuronal resistance to oxidative damage by enhancing cell membrane stability and, in turn, to increase brain antioxidant capacity against oxidative damage [27]. The results of total phenol and flavonoids and antioxidant power of this plant in the present study confirm the high potential of this extract as a natural antioxidant. Also, the results of brain antioxidant capacity test in the present study showed that brain antioxidant capacity decreased in PTZ group and increased in extract treatment groups and this difference was significant at different levels. This result indicates an improvement in brain antioxidant capacity in the kindled rats after receiving the CZ extract. Serum antioxidant capacity was also decreased in PTZ group and increased in extract treatment groups and this difference was significant at different levels. According to the experiments of Srividya et al. 2012 conducted on the hydroalcoholic extract of *Curcuma zedoaria*, total phenol content was 34.45 ± 1.9 mg/g (gallic acid equivalent), and total flavonol content was 45.56 ± 2.38, IC50 value of antioxidant activity for hydroalcoholic extract by DPPH was 930 ± 35.16, antioxidant activity by nitric oxide method equals 1000 < mg/ml and the concentration required for reducing power equivalent to 230.2 ± 1.32 [28]. In 2014, Pyun et al. reported that curcumin has been proposed as a potent antioxidant that reduces the formation of oxygen free radicals and strengthens the antioxidant defense system. It easily crosses the blood–brain barrier so it can be easily accessed by nerve cells and protects them against damaging factors [29].

In the present study, serum and brain MDA levels were higher in PTZ group than in other groups, suggesting seizure and lipid peroxidation damage. Also, in the diazepam, sham and extract treatment groups, MDA levels were decreased compared to PTZ group. In the flumazenil and the effective dose of the extract group, there was an increase in MDA level in compared to the group received effective dose of the extract alone. Guo et al. found that adding curcumin to the culture medium of microglia and cortical neurons reduces oxidative damage by inhibiting lipid peroxidation and also increases the activity of antioxidant enzymes and overall decreased inflammatory response [30].

Although the role of NO in epilepsy has been investigated in a number of studies, there has been no consensus regarding the results [31] and the anticonvulsant [32] and pre-convulsive [33] effects of NO by seizure type, NO source, other neurological transmitters and even sex or age of animals have been reported [34]. In the present study, brain and serum levels of NO were higher in PTZ group and decreased in diazepam, sham and extract groups.

In the present study, it was found that hippocampal tissue changes in the negative control group were significantly different from those in the sham group, which was due to injection of PTZ seizure agent and the changes in the extract receiving groups were slightly less than those in the negative control group. In a study by Kiasalari et al., It was found that oral administration of 100 mg/kg curcumin a week before surgery reduced the toxic effects of kainic acid in the hippocampus so that the severity of seizures decreased. Hippocampal neuronal damage was significantly reduced and neuronal budding in the molecular layer was also attenuated, part of these beneficial effects were achieved by reducing oxidative stress and enhancing the antioxidant defense system [35].

## Conclusion

The results of the present studies confirm the antiepileptic effects of the CZ extract, which is associated with protection against neuronal damage. Further studies are needed to better understand the mechanisms responsible and the precise composition.

## Data Availability

All data and materials of this manuscript are available.

## References

[1] Devi PU, Manocha A, Vohora D (2008). Seizures, antiepileptics, antioxidants and oxidative stress: an insight for researchers. Expert Opin Pharmaco.

[2] Kudin AP, Kudina TA, Seyfried J, Vielhaber S, Beck H, Elger CE (2002). Seizure-dependent modulation of mitochondrial oxidative phosphorylation in rat hippocampus. Eur J Neurosci.

[3] Brooks-Kayal AR, Shumate MD, Jin H, Rikhter TY, Coulter DA (1998). Selective changes in single cell GABA A receptor subunit expression and function in temporal lobe epilepsy. Nat Med.

[4] Welbat JU, Chaisawang P, Chaijaroonkhanarak W, Prachaney P, Pannangrong W, Sripanidkulchai B (2016). Kaempferia parviflora extract ameliorates the cognitive impairments and the reduction in cell proliferation induced by valproic acid treatment in rats. Ann Ana.

[5] Lobo R, Prabhu KS, Shirwaikar A, Shirwaikar A (2009). *Curcuma zedoaria* Rosc.(white turmeric): a review of its chemical, pharmacological and ethnomedicinal properties. J Pharm Pharmacol.

[6] Pohle W, Becker A, Grecksch G, Juhre A, Willenberg A (1997). Piracetam prevents pentylenetetrazol kindling-induced neuronal loss and learning deficits. Seizure.

[7] Ullah HA, Zaman S, Juhara F, Akter L, Tareq SM, Masum EH (2014). Evaluation of antinociceptive, in-vivo & in-vitro anti-inflammatory activity of ethanolic extract of *Curcuma zedoaria* rhizome. BMC Complem Altern M.

[8] Sridhar K, Charles AL (2019). In vitro antioxidant activity of Kyoho grape extracts in DPPH and ABTS assays: estimation methods for EC50 using advanced statistical programs. Food Chem.

[9] Derakhshan Z, Ferrante M, Tadi M, Ansari F, Heydari A, Hosseini MS (2018). Antioxidant activity and total phenolic content of ethanolic extract of pomegranate peels, juice and seeds. Food Chem Toxicol.

[10] Dhir A (2012). Pentylenetetrazol (PTZ) kindling model of epilepsy. Curr Protoc Neurosci..

[11] Tripathi Y, Saini N (2019). Total phenolic, total flavonoid content and antioxidant efficacy of leaves of Eupatorium adenophorum. Int J Pharma Bio Sci.

[12] Peay DN, Saribekyan HM, Parada PA, Hanson EM, Badaruddin BS, Judd JM (2020). Chronic unpredictable intermittent restraint stress disrupts spatial memory in male, but not female rats. Behav Brain Res.

[13] Khalili M, Roghani M, Ekhlasi M. The effect of aqueous *Crocus sativus* L. extract on intracerebroventricular streptozotocin-induced cognitive deficits in rat: a behavioral analysis. Iran J Pharm Res. 2009;8(3):185–91.

[14] Ghiasi S, Vaezi GH, Keramati K. Effects of intracerebroventricular injection of alcoholic extract of Hypericum Perforatum on fear behavior in presence pentylenetetrazole (PTZ) in adult male rat. J Ilam Uni Med Sci. 2010;17(4):36–44.

[15] Cusack CL, Annis RP, Kole AJ, Deshmukh M (2014). Neuronal death mechanisms in development and disease. Cell Death.

[16] Hao F, Jia L-H, Li X-W, Zhang Y-R, Liu X-W (2016). Garcinol upregulates GABAA and GAD65 expression, modulates BDNF-TrkB pathway to reduce seizures in pentylenetetrazole (PTZ)-induced epilepsy. Med Sci Monit.

[17] Duarte FS, Marder M, Hoeller AA, Duzzioni M, Mendes BG, Pizzolatti MG (2008). Anticonvulsant and anxiolytic-like effects of compounds isolated from *Polygala sabulosa* (Polygalaceae) in rodents: in vitro and in vivo interactions with benzodiazepine binding sites. Psychopharmacol.

[18] Syu W-J, Shen C-C, Don M-J, Ou J-C, Lee G-H, Sun C-M (1998). Cytotoxicity of curcuminoids and some novel compounds from *Curcuma zedoaria*. J Nat Prod.

[19] Anovadiya AP, Sanmukhani JJ, Vadgama VK, Tripathi C (2013). Evaluation of antiepileptic and memory retention activity of curcumin per se and incombination with antiepileptic drugs. Asian J Pharm Clin Res.

[20] Nassiri-Asl M, Mortazavi S-R, Samiee-Rad F, Zangivand A-A, Safdari F, Saroukhani S (2010). The effects of rutin on the development of pentylenetetrazole kindling and memory retrieval in rats. Epilepsy Behav.

[21] Uzum AK, Salman S, Telci A, Boztepe H, Tanakol R, Alagol F (2010). Effects of vitamin D replacement therapy on serum FGF23 concentrations in vitamin D-deficient women in short term. Eur J Endocrinol.

[22] Spencer JP (2009). Flavonoids and brain health: multiple effects underpinned by common mechanisms. Genes Nutr.

[23] Scaini S, Ogliari A, Eley TC, Zavos HM, Battaglia M (2012). Genetic and environmental contributions to separation anxiety: a meta-analytic approach to twin data. Depress Anxiety.

[24] Yadav P, Kumar A, Mahour K, Vihan V (2010). Phytochemical analysis of some indigenous plants potent against endoparasite. J Adv Lab Res Biol.

[25] Wilcox KS, Gee JM, Gibbons MB, Tvrdik P, White JA (2015). Altered structure and function of astrocytes following status epilepticus. EPILEPSY BEHAV.

[26] Karalija A, Novikova LN, Kingham PJ, Wiberg M, Novikov LN (2012). Neuroprotective effects of N-acetyl-cysteine and acetyl-L-carnitine after spinal cord injury in adult rats. PLoS ONE.

[27] Nazıroğlu M, Kutluhan S, Uğuz AC, Çelik Ö, Bal R, Butterworth PJ (2009). Topiramate and vitamin E modulate the electroencephalographic records, brain microsomal and blood antioxidant redox system in pentylentetrazol-induced seizure of rats. J Membrane Biol.

[28] Srividya A, Dhanabal S, Yadav AK, Kumar S, Vishnuvarthan V (2012). Phytopreventive anti-hyperlipidemic activity of *Curcuma zedoaria*. Bull Pharm Res.

[29] Pyun G, Yun U, Ryu KH (2014). Efficient frequent pattern mining based on linear prefix tree. Knowl-Based Syst.

[30] Guo JY, Xia B, White E (2013). Autophagy-mediated tumor promotion. Cell.

[31] Wojtal K, Gniatkowska-Nowakowska A, Czuczwar SAJ (2003). Is nitric oxide involved in the anticonvulsant action of antiepileptic drugs. Pol J Pharmacol..

[32] Theard MA, Baughman VL, Wang Q, Pelligrino DA, Albrecht RF (1995). The role of nitric oxide in modulating brain activity and blood flow during seizure. NeuroReport.

[33] Faghir-Ghanesefat H, Keshavarz-Bahaghighat H, Rajai N, Mokhtari T, Bahramnejad E, Roodsari SK (2019). The possible role of nitric oxide pathway in pentylenetetrazole preconditioning against seizure in mice. J Mol Neurosci.

[34] Bahramnjead E, Roodsari SK, Rahimi N, Etemadi P, Aghaei I, Dehpour AR (2018). Effects of modafinil on clonic seizure threshold induced by pentylenetetrazole in mice: involvement of glutamate, nitric oxide, GABA, and serotonin pathways. Neurochem Res.

[35] Kiasalari Z, Roghani M, Khalili M, Rahmati B, Baluchnejadmojarad T (2013). Antiepileptogenic effect of curcumin on kainate-induced model of temporal lobe epilepsy. Pharm Biol.

